# Pancreatic cancer: disease dynamics, tumor biology and the role of the microenvironment

**DOI:** 10.18632/oncotarget.24019

**Published:** 2018-01-06

**Authors:** Daniel Ansari, Helmut Friess, Monika Bauden, Johan Samnegård, Roland Andersson

**Affiliations:** ^1^ Department of Surgery, Clinical Sciences Lund, Lund University, Skåne University Hospital, Lund, Sweden; ^2^ Department of Surgery, Technical University of Munich, Munich, Germany

**Keywords:** pancreatic cancer, disease dynamics, tumor biology, microenvironment

## Abstract

Pancreatic cancer is known for its propensity to metastasize. Recent studies have challenged the commonly held belief that pancreatic cancer is a stepwise process, where tumor cells disseminate late in primary tumor development. Instead it has been suggested that pancreatic tumor cells may disseminate early and develop independently and in parallel to the primary tumor. Circulating tumor cells can be found in most patients with pancreatic cancer, even in those with localized stage. Also, recent phylogenetic analyses have revealed evidence for a branched evolution where metastatic lineages can develop early in tumor development. In this Review, we discuss current models of pancreatic cancer progression and the importance of the tumor microenvironment, in order to better understand the recalcitrant nature of this disease.

## INTRODUCTION

Pancreatic cancer is a devastating disease with a high mortality rate. Due to late symptoms, the vast majority of patients are deemed inoperable at the time of diagnosis as a consequence of locally advanced tumors or distant metastases. The 5-year survival rate is less than 5%, and even in patients who undergo surgical resection, the prognosis is poor and recurrence of the disease is common [1]. Despite advances in the understanding of genetic and epigenetic alterations involved in pancreatic tumorigenesis [2–6], the diagnosis and therapy of this disease still remain an unmet health care need.

Most deaths due to pancreatic cancer are due to metastatic disease. In such a scenario, understanding pancreatic cancer progression becomes important. This has resulted in two fundamental models of metastatic progression. The traditional model places the genetic development of metastatic founder cells within the primary tumor. Disseminated tumor cells are thought to appear late in tumor development [7]. A recent model, on the other hand, suggests early dissemination of cancer cells and independent progression of metastases [8].

Common to both models is the emphasis on the tumor microenvironment, both locally and at ectopic sites. The pancreatic tumor microenvironment is comprised of tumor cells, and a variety of stromal or non-malignant cells including stellate cells [9–11], inflammatory and immune cells [12–14], as well as blood vessels [15–17], extracellular matrix proteins [18–20] and tumor-derived exosomes [21, 22]. It is created and shaped during tumor progression due to the tumor-host interface, leading to a unique microecology that plays a major role in tumor growth, metastasis and response to therapy [23].

Here, we review recent advances in the pathology and molecular genetics of pancreatic cancer, shedding new light on pancreatic cancer progression and factors contributing to disease aggressiveness. A better understanding of pancreatic cancer development and progression may ultimately lead to more effective diagnostic and therapeutic strategies.

## DYNAMICS OF PANCREATIC CANCER PROGRESSION

Metastasis is the dissemination and growth of neoplastic cells in an organ distinct from that in which they originated. A number of steps are required to take place for metastasis to occur, including the ability of cancer cells to reach and survive in the bloodstream, and settle in distant organs. Only a small fraction of the cancer cells that reach the bloodstream are able to form metastases. In order to do so they must be able to interact with the new microenvironment of the distant organ and adapt to the pre-metastatic niche [24, 25].

**Figure 1:**
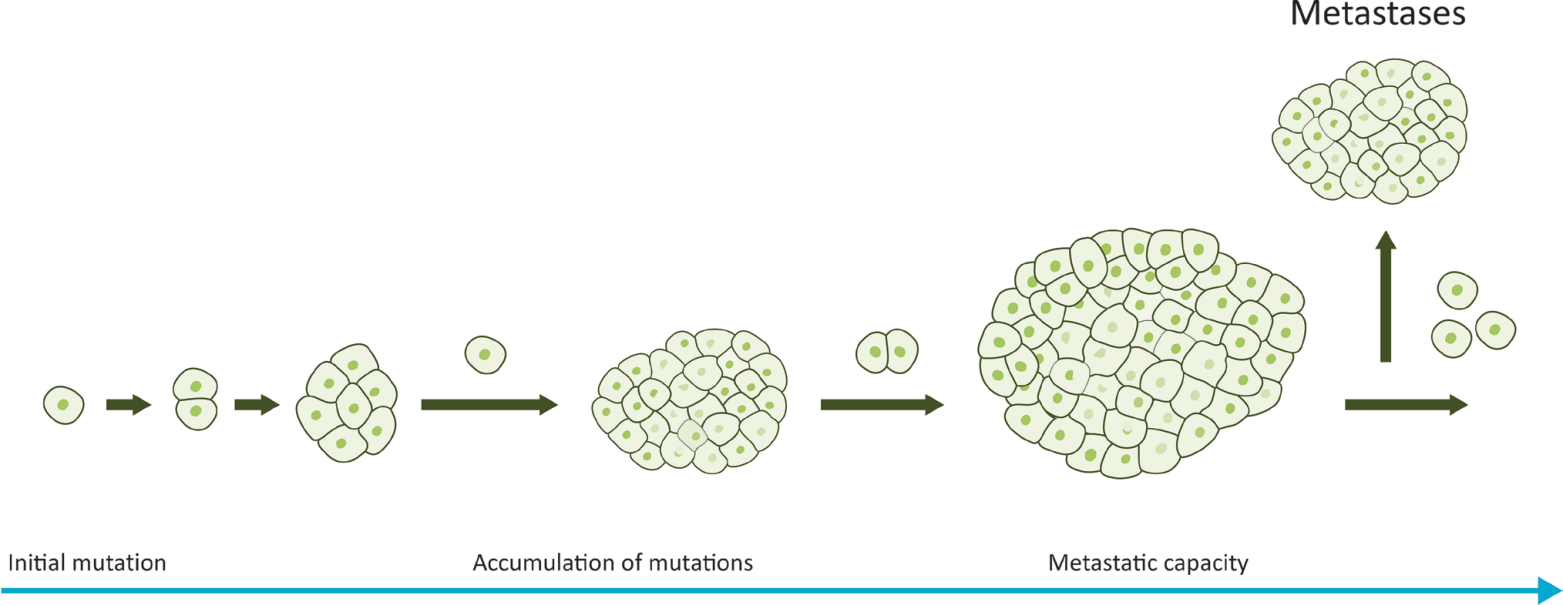
Traditional model of pancreatic cancer progression In the linear progression model accumulation of genetic and epigenetic changes occurs over a long period of time and metastases develop in a late phase when the tumor has reached a detectable size.

The common view of pancreatic cancer pathogenesis has long been a stepwise progression into metastatic disease, as depicted in Figure 1. Pancreatic cancer has been suggested to be an evolutionary disease, obeying the principles of Darwinian evolution. The progression of pancreatic cancer is divided into three major evolutionary stages: i) a driver gene mutation leading to tumor initiation, ii) clonal expansion and iii) dissemination of malignant cells into the microenvironment of the primary tumor and distant sites [26]. In this conceptual model, distant metastasis is the final step in the evolution of pancreatic cancer [7].

**Figure 2:**
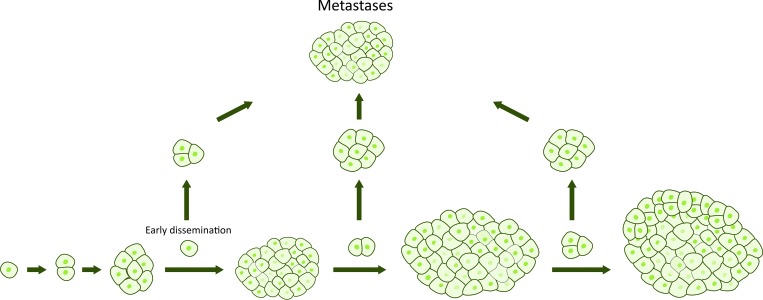
Alternative model of pancreatic cancer progression Tumor cells disseminate at any point of tumor progression and develop independently into metastases in parallel to the primary tumor.

**Figure 3:**
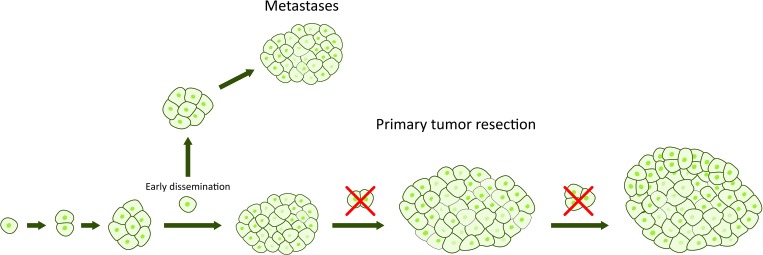
Early resection of the primary tumor without detectable metastases prevents further tumor growth and thereby further tumor dissemination and prolongs survival Unfortunately, not even a radical surgery prevents development of potential metastases from early disseminated cancer cells.

During recent years, however, a new model of the metastatic process in pancreatic cancer has gained interest. According to this model, high metastatic capability might actually be a native feature of some pancreatic tumors, challenging the linear progressive model [8, 27]. The parallel model suggests that tumor cells metastasize early during the tumorigenesis, and continue to evolve independently at distant sites (Figure 2). Of note, circulating tumor cells have been found in most patients with pancreatic cancer, even when the disease was classified as localized by current staging systems [28–30]. Furthermore, it has been found that metastatic lineages can occur early in tumor development and develop from divergent lineages within the primary tumor [31]. What are the consequences of the parallel progression model? It means that not even radical surgery prevents development of potential metastases from early disseminated cancer cells (Figure 3). Until we have the necessary tools to detect the disease before it becomes invasive, pancreatic cancer must be viewed upon and treated as a systemic disease at the time of diagnosis.

## TUMOR BIOLOGY

The current TNM staging system is the gold standard for determining prognosis and directing treatment in pancreatic cancer. The size of the primary tumor has a fundamental impact on staging. However, previous studies have shown that even the smallest of tumors are able to metastasize. The relationship between the primary tumor size and distant metastatic rates and survival was recently evaluated in a large population-based study [32]. A total of 58,728 patients with pancreatic cancer from the Surveillance, Epidemiology and End Results (SEER) database were analyzed. It was found that the rate of distant metastasis increased in a non-linear fashion with increasing size of the primary tumor. Importantly, these data showed that as much as one third of the minimal tumors (1-5 mm) already were associated with distant metastases. Clearly, pancreatic cancer is a heterogeneous disease, and some tumors progress rapidly, while others behave in a more indolent fashion [33]. Many attempts have been made to subclassify pancreatic tumors at the molecular level [2, 34, 35], but still no subclassification has yet received clinical translation.

## THE MICROENVIRONMENT

**Figure 4:**
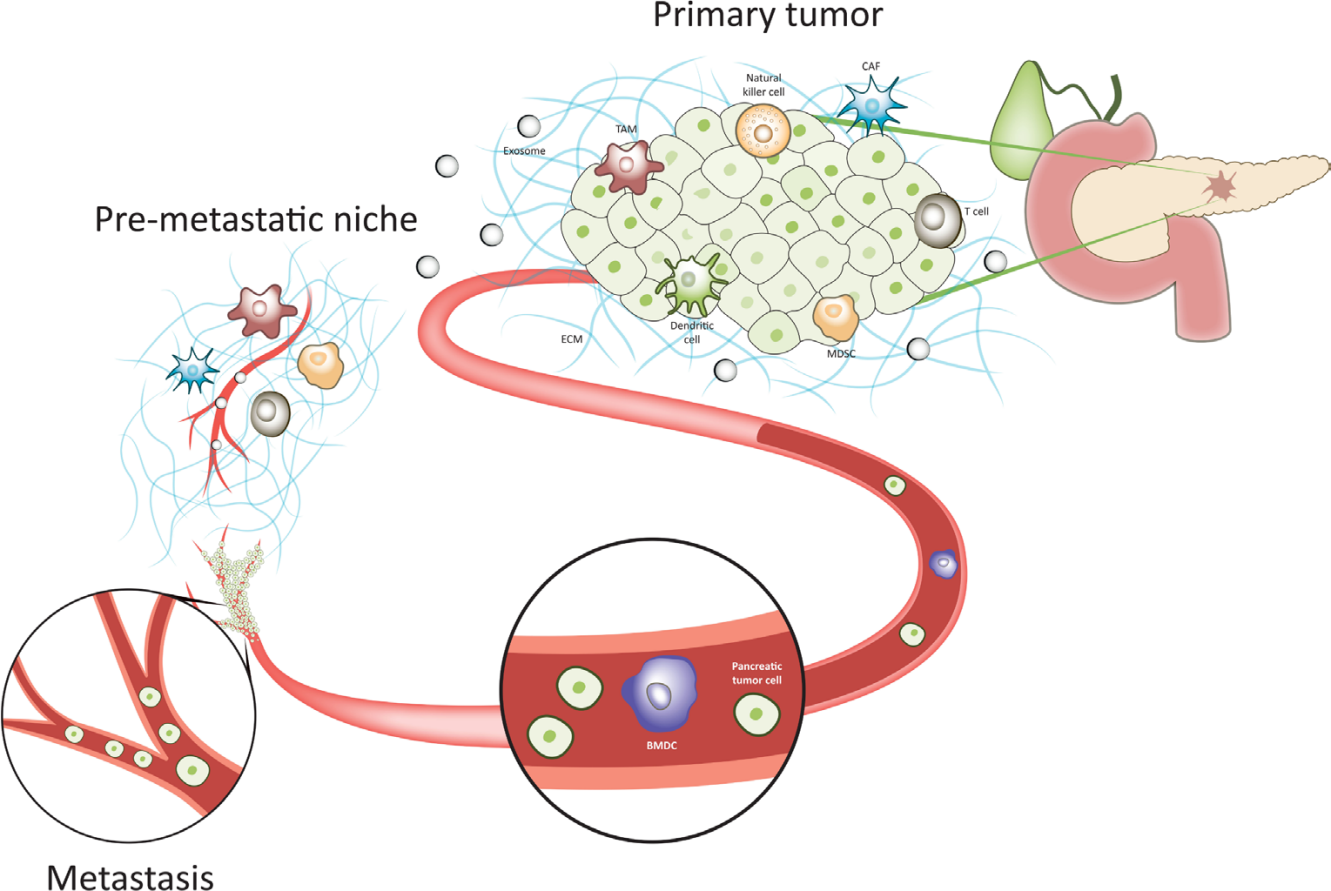
The microenvironment regulates pancreatic cancer progression and metastasis Tumor-derived exosomes, bone marrow-derived cells and local stromal components promote metastasis by inducing pre-metastatic niches in distant organs, which are conducive to the survival and outgrowth of tumor cells before their arrival at these sites. BMDC: bone marrow-derived cell; CAF: cancer-associated fibroblast; ECM: extracellular matrix; MDSC: myeloid-derived suppressor cell; TAM: tumor-associated macrophage.

The tumor microenvironment plays a key role in the development and progression of pancreatic cancer. The microenvironment is comprised of tumor cells, non-tumor cells, extracellular matrix, cytokines, growth factors, and exosomes that regulate autocrine, paracrine and endocrine communication, affecting tumor progression (Figure 4). Unlike the cancer cells, the stromal cells are genetically stable and represent a potential therapeutic target [36]. However, stromal re-programming rather than eradication *per se* may be more effective based on previous experimental work [37].

### Tumor-initiating cells

The cancer stem cell theory proposes that solid tumors contain a small subpopulation of tumor-initiating cells or cancer stem cells that are responsible for tumor initiation, progression and chemoresistance [38–40]. The identification of pancreatic cancer stem cells has been based on their increased tumorigenic potential in immunocompromised mice [41]. However, it has been proposed that only a subset of cancer stem cells have the ability to metastasize. A subpopulation of CD133+ CXCR4+ pancreatic cancer stem cells was been identified at the tumor invasive front. Elimination of these cells inhibited metastasis formation [42]. On the other hand, it has been suggested that all tumor cells are biologically homogenous and have the potential to promote tumor initiation, growth and progression [43]. Tumorigenesis has been suggested to occur in normal somatic cells as a result of stochastic events caused by intrinsic factors such as genomic instability and extrinsic factors originating from the tumor microenvironment.

### Epithelial-mesenchymal transition

Local invasion is facilitated by changes in the shape and function of tumor cells. This epithelial–mesenchymal transition (EMT) is a profound feature of pancreatic cancer that occurs already in the very first stages of tumor development [44]. It implies changes in the adhesion molecules expressed by the cell, with acquisition of a migratory and invasive phenotype, which favors cellular disassociation, degradation of the basement membrane and ultimately contribute to early dissemination and drug resistance [45, 46]. The process of EMT is characterized by downregulation of epithelial markers (e.g. E-cadherin) and upregulation of mesenchymal markers (e.g. N-cadherin, vimentin and fibronectin) [47]. Several transcription factors, such as Snai1, Slug and Twist1, are involved in activating EMT programs in pancreatic cancer [48]. According to recent data, inflammation is a major driver of EMT in pancreatic cancer cells [49]. While EMT promotes dissemination of cancer cells, it has also been found that metastases show an epithelial histology. The reverse process, i.e. mesenchymal-epithelial transition (MET), is believed to induce the epithelial phenotype at distant sites [50].

### Cancer-associated fibroblasts

Pancreatic stellate cells are a subset of pancreatic cancer-associated fibroblasts. The stellate cells have many functions in the normal pancreas, such as maintenance of normal tissue architecture through regulation of extracellular matrix turnover, as well as immunological functions [51, 52]. In pancreatic cancer, stellate cells change morphology into a myofibroblast-like cell, which is characterized by alpha-smooth muscle actin expression and induction of a fibroinflammatory response, including excessive production of extracellular matrix proteins, growth factors and cytokines. Accumulating evidence suggest that stellate cells play a central role in pancreatic tumor invasion and progression. Interestingly, it has been reported that pancreatic stellate cells support tumor metabolism and growth through the secretion of non-essential amino acids, importantly alanine [11]. In experimental models of pancreatic cancer, the reversion of activated stellate cells to their quiescent state has shown anti-tumor effects. All-trans retinoic acid (ATRA) has been found to reprogram the pancreatic stellate cells to a more quiescent phenotype through the RAR-β/MLC-2 pathway, which downregulates contractility and mechanosensing in the stellate cells, leading to reduced migration, less desmoplasia and suppression of cancer invasion [53]. However, more research is needed into studying stellate cells, not only in pancreatic cancer, but also under physiological conditions in healthy tissue, in order better understand normal function and pathological activation.

### Inflammation

Inflammatory processes are highly involved in pancreatic cancer pathogenesis [54]. In the clinical setting, a pronounced inflammatory response has been associated with disease progression and poor survival in patients with manifest pancreatic cancer [55–58]. Much effort has been put into elucidating the underlying mechanisms that contribute to inflammation-induced tumorigenesis and potential ways to attenuate this process. It has been suggested that the presence of inflammation cooperates with the Kras oncogene to drive pancreatic cancer progression, while mutant Kras alone is not sufficient to drive pancreatic tumorigenesis [59]. Recent work suggests that the fibroinflammatory response, induced by pancreatic stellate cells, can influence the epigenome and metabolome in pancreatic cancer cells, including downstream Kras targets (e.g. Csf2, Rrm2, Sc4mol) [60]. Some potential paracrine factors of interest include connective tissue growth factor (CTGF), hepatocyte growth factor (HGF), insulin-like growth factors (IGFs) and interleukin-6 (IL-6) involved in Ras-MAPK, MYC and STAT3 signaling.

### Immunity

Pancreatic cancer is characterized by an immunosuppressive microenvironment [61, 62]. Key regulators of the host tumor immune response include T cells, natural killer cells, macrophages, myeloid derived suppressor cells and dendritic cells [13]. Furthermore, pancreatic tumors actively recruit bone marrow-derived cells, which contribute to neovascularization and establishing the pre-metastatic niche [63–65]. The study of long-term survivors has provided valuable insights into the heterogeneous immunological profile of pancreatic cancer [66]. Interestingly, the tumors of long-term survivors displayed higher amounts of CD8+ T cells, cytolytic CD8+ cells, regulatory T cells, mature dendritic cells and macrophages, while the numbers of of CD4+ T cells were reduced. The long-term survivors had enhanced neoantigen quality (including MUC16) and neoantigens with homology to infectious disease-derived peptides.

### Exosomes

Exosomes are extracellular vesicles with a size of 40–100 nm that provide a means of intercellular communication [67]. They may contain DNA fragments, mRNAs, miRNAs, proteins and lipids. Evidence suggests that tumor-derived exosomes can shape the tumor microenvironment by facilitating recruitment and reprogramming of individual stromal components [68–70]. It has been reported that pancreatic tumor-derived exosomes induce liver pre-metastatic niche formation in experimental models of pancreatic cancer [21]. The exosomes were found to target and activate Kupffer cells, induce fibrotic pathways, leading to recruitment of bone marrow-derived cell migration to the liver. Depletion of macrophage migration inhibitory factor (MIF) in exosomes inhibited pre-metastatic niche formation and liver metastasis.

## CONCLUSIONS

The lack of progress in the management of pancreatic cancer by traditional methods (i.e. surgery, conventional chemotherapy, radiation) puts great hope into translational research to explore new strategies to change the dire course of the disease. Clinical and molecular data suggest that pancreatic cancer develops as a consequence of parallel progression and therefore must be viewed upon as a systemic disease even in the earliest stages of cancer development. As metastases can arise early in tumor development, research efforts should be directed towards a better understanding of fundamental drivers of tumorigenesis and better characterization of primary and metastatic lesions. More research should be directed towards understanding the tumor microenvironment, including its immunosuppressive role and the potentials for therapeutic targeting.
